# Deep‐Learning‐Based Preprocessing for Quantitative Myocardial Perfusion MRI

**DOI:** 10.1002/jmri.26983

**Published:** 2019-11-11

**Authors:** Cian M. Scannell, Mitko Veta, Adriana D.M. Villa, Eva C. Sammut, Jack Lee, Marcel Breeuwer, Amedeo Chiribiri

**Affiliations:** ^1^ School of Biomedical Engineering and Imaging Sciences King's College London UK; ^2^ Alan Turing Institute London UK; ^3^ Department of Biomedical Engineering, Medical Image Analysis group Eindhoven University of Technology Eindhoven The Netherlands; ^4^ Bristol Heart Institute and Translational Biomedical Research Centre, Faculty of Health Science University of Bristol UK; ^5^ Philips Healthcare, Best The Netherlands

**Keywords:** quantitative myocardial perfusion, convolutional neural networks, machine learning, automated image analysis

## Abstract

**Background:**

Quantitative myocardial perfusion cardiac MRI can provide a fast and robust assessment of myocardial perfusion status for the noninvasive diagnosis of myocardial ischemia while being more objective than visual assessment. However, it currently has limited use in clinical practice due to the challenging postprocessing required, particularly the segmentation.

**Purpose:**

To evaluate the efficacy of an automated deep learning (DL) pipeline for image processing prior to quantitative analysis.

**Study Type:**

Retrospective.

**Population:**

In all, 175 (350 MRI scans; 1050 image series) clinical patients under both rest and stress conditions (135/10/30 training/validation/test).

**Field Strength/Sequence:**

3.0T/2D multislice saturation recovery T_1_‐weighted gradient echo sequence.

**Assessment:**

Accuracy was assessed, as compared to the manual operator, through the mean square error of the distance between landmarks and the Dice similarity coefficient of the segmentation and bounding box detection. Quantitative perfusion maps obtained using the automated DL‐based processing were compared to the results obtained with the manually processed images.

**Statistical Tests:**

Bland–Altman plots and intraclass correlation coefficient (ICC) were used to assess the myocardial blood flow (MBF) obtained using the automated DL pipeline, as compared to values obtained by a manual operator.

**Results:**

The mean (SD) error in the detection of the time of peak signal enhancement in the left ventricle was 1.49 (1.4) timeframes. The mean (SD) Dice similarity coefficients for the bounding box and myocardial segmentation were 0.93 (0.03) and 0.80 (0.06), respectively. The mean (SD) error in the RV insertion point was 2.8 (1.8) mm. The Bland–Altman plots showed a bias of 2.6% of the mean MBF between the automated and manually processed MBF values on a per‐myocardial segment basis. The ICC was 0.89, 95% confidence interval = [0.87, 0.90].

**Data Conclusion:**

We showed high accuracy, compared to manual processing, for the DL‐based processing of myocardial perfusion data leading to quantitative values that are similar to those achieved with manual processing.

**Level of Evidence:** 3

**Technical Efficacy Stage:** 1

J. Magn. Reson. Imaging 2020;51:1689–1696.

FIRST‐PASS MYOCARDIAL PERFUSION IMAGING with cardiac magnetic resonance imaging (MRI) has been shown to be highly accurate for the detection of coronary artery disease (CAD)^1^, ^2^ and suitable for guiding the management of patients with an intermediate risk of CAD.^3^, ^4^ Visual interpretation of the images, however, is complex, time‐consuming, and the accuracy of the results is dependent on the level of training and experience of the operator, thereby limiting the adoption of these techniques outside highly experienced centers.^5^

An alternative to the visual assessment is quantitative perfusion analysis, which is made possible by the use of tracer‐kinetic modeling.^6^ Quantitative perfusion analysis can be automated^7^, ^8^ leading to fast, robust, and reproducible estimates of myocardial perfusion.^9^ Quantitative analysis has been validated against positron emission tomography (PET),^10^, ^11^, ^12^ fractional flow reserve,^13^ and microspheres.^14^, ^15^ Sammut et al have also recently demonstrated the independent prognostic value of quantitative stress perfusion MRI in patients with suspected CAD.^16^ The availability of automated and standardized methods for quantitative analysis could facilitate the wider adoption of first‐pass myocardial perfusion imaging.

The quantitative analysis requires challenging image processing.^17^ It is required to identify the left ventricular blood pool to extract an arterial input function (AIF) to use along with the myocardial tissue curves in the model fitting. The segmentation of the myocardium is also desirable, as it allows the analysis of values specifically in the region of interest (ROI) and the computation of the myocardial perfusion reserve (MPR), which is the ratio of perfusion values at stress to the values at rest. The use of a segmentation also requires fewer voxels to be fit to the model, which is more time‐efficient and allows the use of more advanced fitting algorithms that take advantage of spatial information.^18^, ^19^ Further advanced analysis techniques involve the assessment of the transmural gradient in contrast uptake across the myocardium^20^ or the assessment of the temporal dyssynchrony of first‐pass perfusion,^21^ for which an ROI is necessary. The identification of the right ventricular (RV) insertion points would also be beneficial in order to divide the myocardial segmentation into the standard American Heart Association (AHA) segments^22^ and to relate perfusion abnormalities to coronary territories.

Myocardial perfusion image series present unique challenges to automated segmentation approaches due to the dynamic contrast‐enhancement and the relatively low signal‐to‐noise ratio (SNR). We propose that the automation of these processing steps can be achieved by leveraging the power of machine learning. In particular, deep learning has produced impressive results in many computer vision tasks such as image detection and recognition. Recently, deep learning has also seen more attention in the field of medical image analysis^23^ and specifically in cardiac MR image analysis with fully convolutional neural networks (FCNs) being applied to the segmentation of anatomical structures in a variety of different applications.^24^, ^25^

In this work, we developed deep‐learning models in order to achieve the requisite preprocessing steps prior to quantitative modeling. These steps were tested individually and as part of the fully‐automated pipeline.

## Materials and Methods

### *Subjects*

The dataset consisted of 175 subjects (64.3 ± 10.3 years old; 136 male) with suspected CAD referred on a clinical basis to King's College London Cardiac MR Service at St Thomas' Hospital (Guy's and St Thomas' NHS Trust). The dataset was randomly split into three sets of 135/10/30 for training/validation/testing. The full demographic and clinical characteristics of the patients is reported in the Supplementary Material, Table S1. The study was conducted in accordance with the Declaration of Helsinki (2000) and was approved by the National Research Ethics Service (15/NS/0030). All patients provided written informed consent.

### *Imaging*

All examinations were performed with a 3T system (Achieva TX, Philips Healthcare, Best, The Netherlands) using a 32‐channel cardiac phased array receiver coil. Perfusion images were acquired in three left ventricle (LV) short‐axis slices (apical, mid‐cavity, and basal) at mid‐expiration with a saturation‐recovery gradient echo method (repetition time / echo time 3.0/1.0 msec, flip angle 15°, saturation‐recovery delay 120 msec, 5‐fold *k‐t* sensitivity encoding [*k‐t* SENSE] acceleration with 11 training profiles, giving a net acceleration of 3.8‐fold, spatial resolution 1.2 × 1.2 × 10 mm^3^). Stress images were acquired during adenosine‐induced hyperemia (140 μg/kg/min); 0.075 mmol/kg of bodyweight gadolinium (Gd) extracellular contrast agent (gadobutrol, Gadovist, Bayer, Germany) was injected at 4 mL/s followed by a 20‐mL saline flush for each perfusion acquisition. Each bolus of gadobutrol was preceded by a diluted prebolus with 10% of the dose to allow quantification of perfusion, according to published methods.^26^

### *Processing Pipeline*

**Figure 1:**
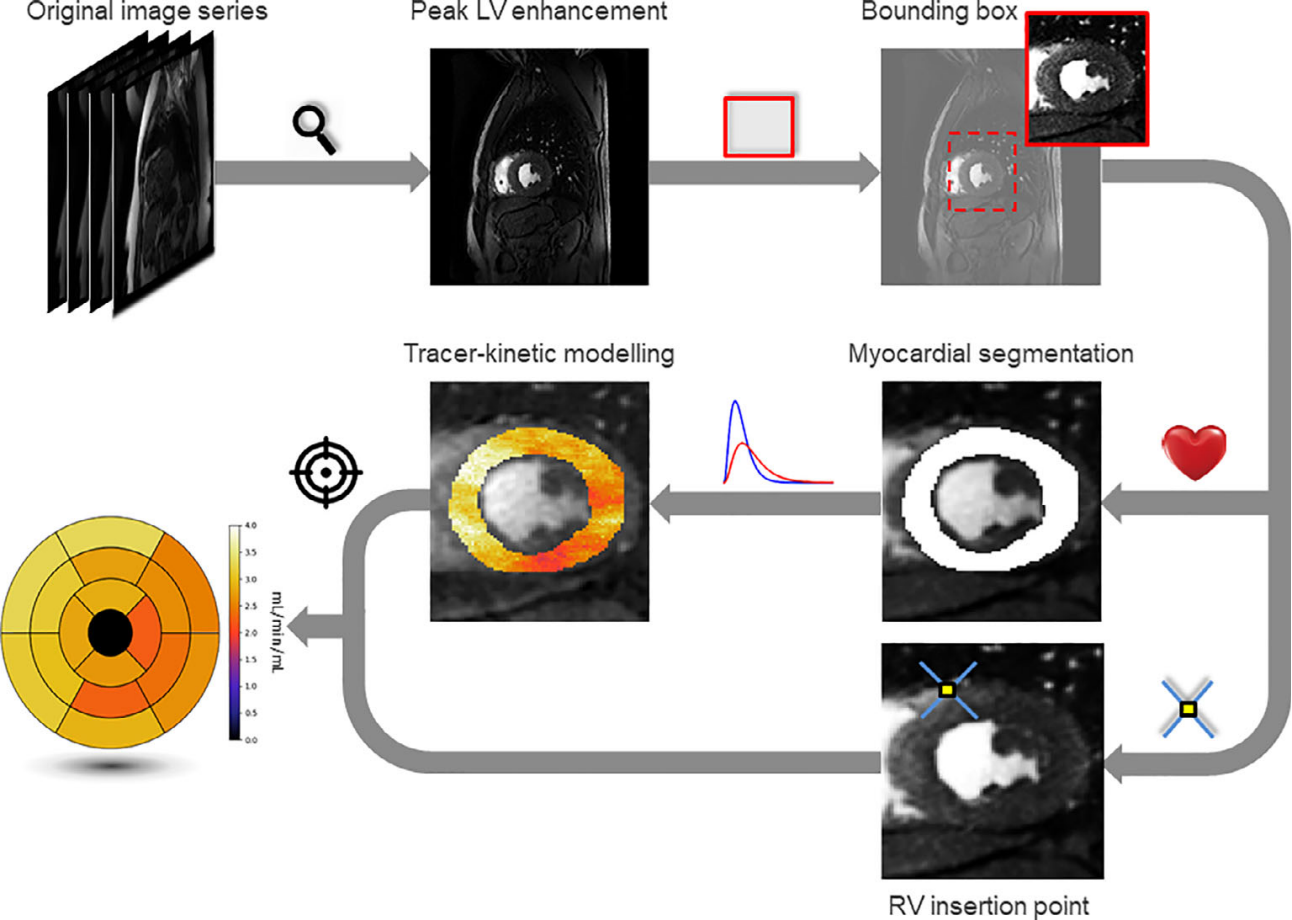
The flow chart representing the pipeline for automated myocardial perfusion quantification. The peak LV enhancement frame in the image series is first identified. This timeframe is then cropped (motion correction is then applied), the myocardium segmented, and RV insertion point determined. Perfusion is quantified using tracer‐kinetic modeling in the myocardium and this is combined with the RV insertion point to generate the bullseye plot.

As shown in Fig. 1, the first step of the pipeline is to detect the timeframe from the image series that corresponds to peak signal enhancement in the LV. Using this image, a bounding box is detected that encompasses the LV cavity and LV myocardium. The cropped image series are then passed to the motion correction scheme that we have described in detail in previous work.^27^ The next step involves segmenting the motion‐corrected and cropped peak LV contrast‐enhancement timeframe to generate a myocardial mask and then the RV insertion points are detected. The AIF is extracted from a region identified using a region‐growing algorithm starting from the position of highest signal inside the endocardial boundary, as defined by the automated segmentation. The AIF along with the voxelwise concentration curves extracted from the myocardium are then used for perfusion quantification using tracer‐kinetic modeling. The RV insertion points are used to relate the quantitative perfusion values to AHA 16‐segment model. The full pipeline proposed in this section is summarized in Fig. 1.

Each constituent component of the pipeline was evaluated using a suitable metric. Furthermore, the quantitative perfusion values achieved with the fully automated pipeline were then compared to those achieved using the manual analysis from an expert operator. This allows the assessment of the effectiveness of the whole pipeline and demonstrates the feasibility of its unsupervised deployment in the clinic.

### *Training Labels*

The epicardial and endocardial borders were manually traced at the time of peak LV enhancement using cvi^42^ software (Circle Cardiovascular Imaging, Calgary, Alberta, Canada) by an experienced operator (E.S., level 3 competency accreditation,^28^ with more than 5 years of experience in cardiac MRI). The RV insertion points were subsequently marked. The timepoint was found by scrolling through the timeframes in the viewer until a satisfactory frame was reached and this timepoint was used for training the LV peak enhancement classifier. The training labels for the bounding box were obtained from the segmentation by computing the smallest box that fits the entire myocardium and expanding it by 20 voxels in each dimension. This analysis was repeated by a second experienced operator (A.V., level 3 accredited^28^ with more than 5 years of experience in cardiac MRI) for the test set to assess the interobserver variability rate.

### *Training Details*

Each of the 175 patients in the dataset underwent perfusion imaging under both rest and stress conditions in which three LV short‐axis slices were acquired yielding a total of 1050 (three imaging planes at both rest and stress for each patient) individual image series. The networks were trained individually for each of the four steps. Prior to training, the images were interpolated to the required dimension, as described in the individual sections, using bicubic interpolation. All images were normalized to have intensity values in the range of [0,1]. On‐the‐fly data augmentation was applied to the training images, which consists of applying random amounts of translation, rotation, scaling, intensity variation, and noise to the images. A batch size of 32 was used in the training of all networks. L2 regularization on the parameters of the convolution kernels was used with a weight of 0.001. The respective cost functions were optimized using the Adam optimizer^29^ with a learning rate of 0.0001 until convergence. Early stopping with a patience of 3000 iterations, assessed using the validation accuracy, was used to determine convergence.

### *Peak LV Enhancement Detection*

A convolutional neural network (CNN) was used to identify the timeframe corresponding to peak contrast‐enhancement in the LV. The CNN takes each timeframe in the image series (256 × 256 voxels) along with the two preceding and two subsequent timeframes as input and outputs as a single number that represents the probability that that timeframe corresponds to the peak LV enhancement in the series. The CNN consists of four convolutional layers followed by two fully‐connected layers and is similar to those previously shown to be successful for image recognition tasks.^30^ Each convolutional layer uses 3 × 3 kernels and is followed by a 2 × 2 max‐pooling layer. It uses batch normalization and rectified linear unit (ReLU) activations except for the output layer, which uses a softmax activation, as shown in the Supplementary Material, Table S2. Dropout is used with probability 0.5 in the fully‐connected layers. The model was trained by minimizing the cross‐entropy loss function.

In order to identify the time of peak LV enhancement in a new image series, the trained classifier was applied individually to each timeframe in the image series. This approach gives a probability for each timeframe to be the peak LV enhancement image. The timeframe with the highest probability is taken as the estimate. A plot of the probability over time for an image series is shown in the Supplementary Material, Fig. S1.

### *Bounding Box Detection*

**Figure 2:**
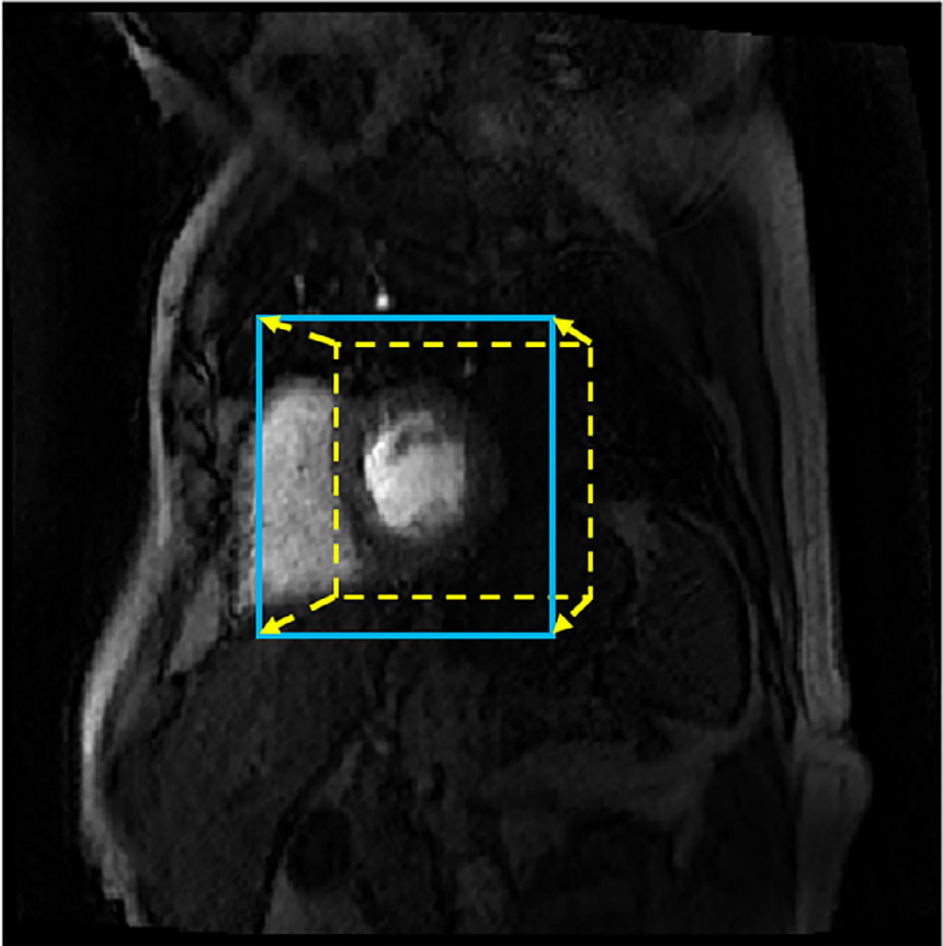
The original proposed ROI (dotted line yellow bounding box) for an example patient. The arrows indicate the deformation output by the CNN to give the ROI for this patient with the detected bounding box shown as the blue continuous line bounding box.

The architecture used to detect the bounding box is the same as that used in the previous step except that the output is now four continuous values rather than the class probabilities (a linear activation is used for the four output units). The network takes the frame of peak LV enhancement as input (256 × 256 voxels) and outputs the parameters that define the bounding box. It is, however, known to be challenging to train a network to directly detect the coordinates of the corners of the bounding box.^31^ A solution to this problem was inspired by the idea of region proposals used by the Faster R‐CNN architecture.^32^ That is, it first assumes that the object, which in this case is the LV cavity and LV myocardium, is within a 75 × 75 voxel ROI centered around the center of the image. The CNN then outputs how much to adjust this ROI so that it better fits the area of interest. The output of the CNN is the displacement of the center of the proposed ROI and scaling factors for the width and height of the proposed ROI. An example image is shown in Fig. 2, with the original proposed ROI and the identified deformation. The mean squared error between the computed transformation of the proposed ROI and the true transformation required was optimized. The CNN was trained using only the peak LV enhancement timeframe from the basal slice and during testing is only applied to the basal slice. Due to the shape of the LV and the planning of the short axis, the bounding box computed on the basal LV slice also applies to the mid‐ventricular and apical slices.

### *Myocardial Segmentation*

The myocardial segmentation utilizes the U‐Net architecture,^33^ which is a fully convolutional network. The input to the network is an image of size 96 × 96 voxels (the cropped and motion‐corrected peak LV enhancement frame) and the output is an image of the same size which corresponds to the voxelwise classifications of the myocardium. The architecture is summarized in the Supplementary Material, Table S3. The cost function that was optimized was the Dice similarity coefficient (DSC)^34^ between the detected segmentation and the human operator segmentation.

The final segmentation is taken as the largest connected component of the binary mask. Failed segmentations are detected automatically by assessing whether the segmentation achieves the expected "closed‐loop" shape of the myocardium. In the case of a failed segmentation, a correction is attempted in a similar manner to Fahmy et al.^25^ As previously described, the nearby timeframes have very similar appearances. Therefore, in the case of a failed segmentation, the segmentation network is applied to all images within two timeframes of the detected peak LV enhancement. The segmentation from the closest timeframe that achieves the expected shape is taken as the segmentation.

### *Insertion Point Detection*

The problem of detecting landmarks in medical images is known to be challenging.^35^ This is due to the high noise levels, large variation in the location of the landmark across subjects, and differences due to subjective positioning of the landmarks by different operators. This makes it extremely difficult to train a regression model to output the coordinates of the landmark. An image‐to‐image approach such as U‐Net can be used to output a segmentation that contains just the one voxel of the landmark location. However, such an approach suffers from the class imbalance problem.

**Figure 3:**
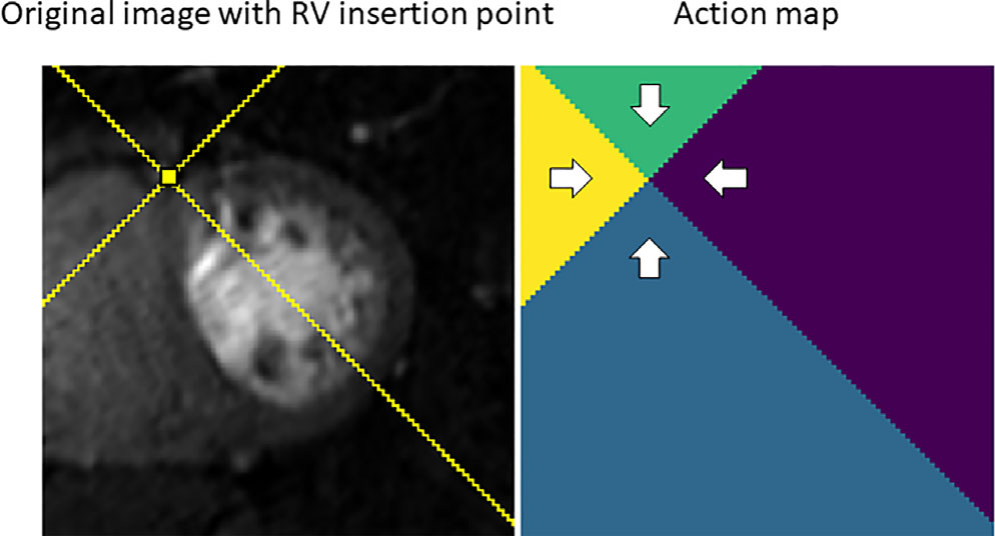
Left: the RV insertion point marked on an example patient with the lines of slope ± 1 that separate the regions of the action map overlaid. Right: The resulting action map, with the direction towards the landmark point shown for each pixel.

Our approach builds on the idea of supervised action classifiers, as proposed by Xu et al.^31^ For each case, an action map is created that represents for each voxel in the image the direction (left, right, up, or down) towards the landmark. An example activation map is shown in Fig. 3 (right). An FCN was then trained to detect which one of these four partitions each voxel belongs to. The U‐Net architecture is used here and is the same as was used for the myocardial segmentation except for the output activation, which is a softmax rather than a sigmoid to reflect the fact that this is now a multiclass classification problem. The cross‐entropy loss function was optimized. From the computed activation maps, regression lines were fit to the boundaries of the partitions and the estimate of the RV insertion point was taken as the intersection of these lines, as shown in Fig. 3 (left).

### *Evaluation*

Each step of the pipeline was evaluated individually by computing a relevant metric for each patient in the test set. For the peak LV enhancement frame detection, the mean difference (in number of timeframes) between the visually chosen timeframe and the detected timeframe was used to evaluate the performance. For both the bounding box detection and the myocardial segmentation steps, the DSC between the outputs and those that were manually acquired is reported. For the segmentation, the metric is compared to the interobserver variability rate found from repeated segmentations by different operators. For the RV insertion points, the Euclidean distances in terms of mm was used to measure the performance.

### *Perfusion Quantification*

Quantitative perfusion analysis was performed on the test cases using both the manually obtained labels and the deep‐learning outputs. The perfusion quantification used a two‐compartment exchange model^6^ for which the kinetic parameters were inferred using hierarchical Bayesian inference, as previously described.^19^ Bland–Altman analysis was used to analyze the bias and limits of agreement between the manual and automated analysis and the linear relationship and intraclass correlation (ICC) between the obtained quantitative values was assessed.

## Results

Representative example cases, with a comparison between manual and automated processing, are shown in Supplemental Figs. S3–S7.

### *Peak LV Enhancement Detection*

The accuracy of the classifier when applied individually to images in the test set was 97.6%. When the peak LV enhancement frame was chosen, as described in the Methods section, the mean (standard deviation [SD]) difference in terms of timeframes (*n* = 60, 30 patients rest and stress) was 1.48 (1.4). The maximum error was three timeframes. It can be noted that even in this case the detected timeframe is very similar to the manual choice and is a reasonable choice for the peak LV enhancement frame, shown in Supplementary Material Fig. S2.

### *Bounding Box Detection*

The mean (SD) DSC between the detected and manually selected bounding box for the test set (*n* = 60, 30 patients rest and stress) was 0.93 (0.03).

### *Myocardial Segmentation*

The mean (SD) DSC between the automated and manual segmentations (*n* = 180, 30 patients with three imaging slices rest and stress) was 0.80 (0.06). The lowest DSC recorded on the test set was 0.69; this image with its corresponding manual and automated segmentation is shown in Fig. S5d. The segmentation of 5 out of 180 test images failed and they were replaced with a successful segmentation computed using a nearby timeframe. The mean (SD) DSC between the segmentations of observer 1 and observer 2 was 0.83 (0.05). Some example images from the different observers are shown in Supplementary Material Fig. S8.

### *RV Insertion Point Detection*

The mean (SD) Euclidean distance (in mm) between the automated and manually chosen RV insertion points (*n* = 360, 180 imaging slices × 2 insertion points) was 2.8 (1.8).

### *Perfusion Quantification*

**Figure 4:**
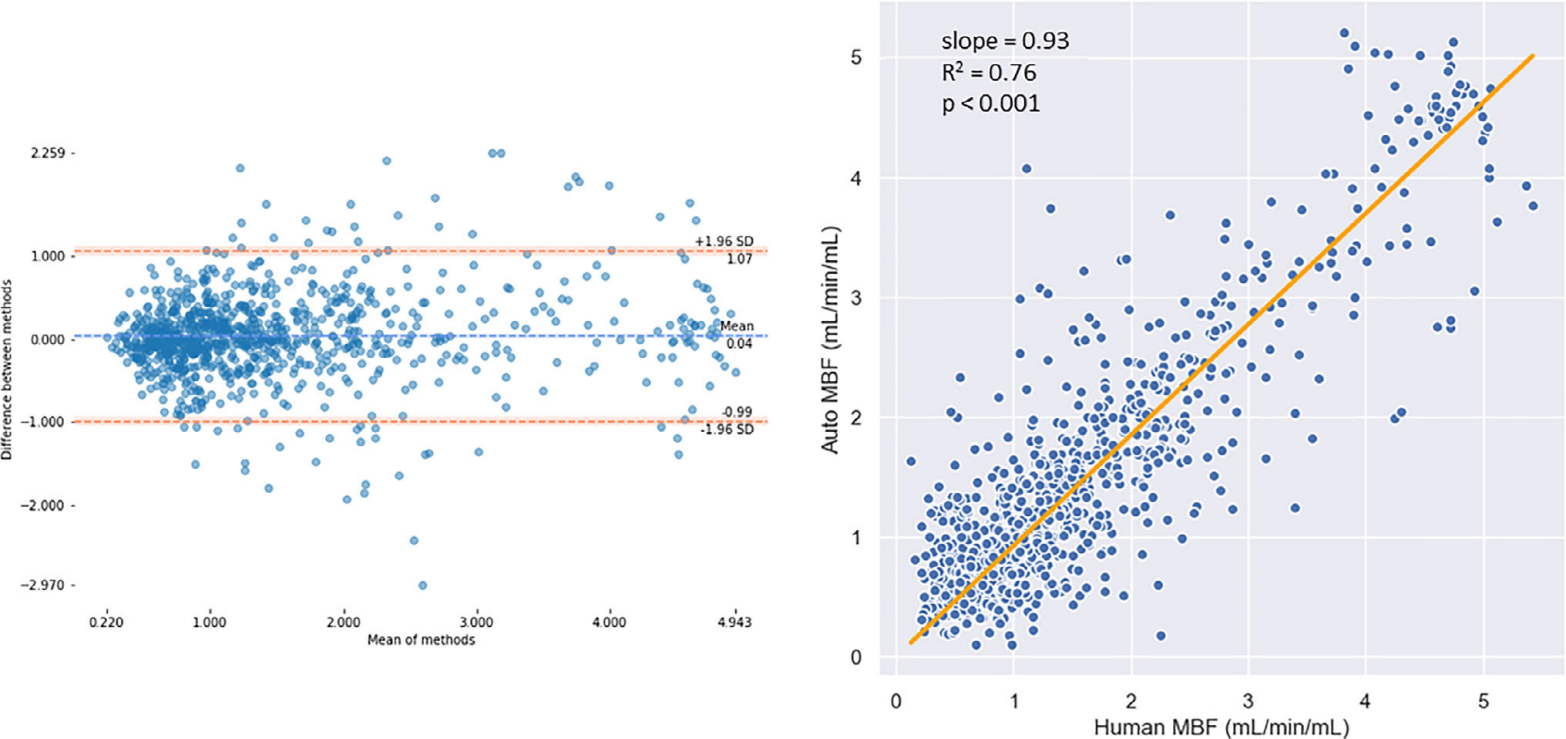
Left: Bland–Altman plots of the automatically processed vs. manually processed quantitative perfusion values averaged over each of the 16 AHA segments. Blue and orange lines represent the bias and ± 1.96 SD limits, respectively, with the shaded regions being the 95% confidence intervals. Right: A scatterplot of the manually processed vs. the automatically processed quantitative perfusion values averaged over each segment of the myocardium. The plotted line is the computed line of best fit with no intercept (slope = 0.93).

The automatically and manually processed test image series resulted in a mean (SD) myocardial blood flow (MBF) of 0.93 (0.37) and 0.91 (0.39) mL/min/mL at rest (*n* = 90) and 2.04 (0.89) and 2.09 (1.26) under stress (*n* = 90), respectively. These values are in line with the ranges previously reported in the literature.^7^, ^8^, ^36^ The use of the RV insertion points further allows the division of the myocardium from the three acquisition slices into the AHA 16‐segment model. The Bland–Altman analysis showed a good agreement between the automated and manual MBF values on a per‐segment basis (*n* = 960, 30 patients with rest and stress × 16 AHA segments) (Fig. 4, left) with the bias being 2.6% of the mean MBF value. There was a strong correlation between the MBF values automatically and manually processed with a slope (with no intercept) on a per‐segment basis of 0.93 with an R^2^ of 0.76 (Fig. 4, right). The ICC was 0.89, 95% confidence interval [0.87, 0.90].

## Discussion

In this work we introduced an automated, deep‐learning‐based preprocessing pipeline for the quantification of myocardial perfusion MRI. The deep learning pipeline processes an image series in a few seconds, compared to roughly 5 minutes for a manual operator, allowing the full quantitative analysis to be performed automatically in just a few minutes. Each step of the pipeline was validated independently, with good results reported. The accuracy of the segmentation was comparable to the interobserver agreement and the quantitative analysis performed with the fully automated pipeline yielded MBF values that were in line with those computed with the manual interaction at each step. The fully automated pipeline was also successful in each image series (180/180) in our test set, indicating the robustness of this approach. As demonstrated by the similarity of the quantitative perfusion values obtained with both the automated and manual pipelines, the pipeline is not sensitive to the errors seen in detecting the peak LV enhancement frame, bounding box, and RV insertion points or segmenting the myocardium.

Despite the increased challenges posed by first‐pass perfusion images, the average (SD) DSC reported is in a similar range to that reported for the segmentation in a comparable automated pipeline for T_1_ mapping (0.80 [0.06] vs. 0.85 [0.07]).^25^ It is also similar to the performance of the model Bai et al^24^ developed when applied to a clinical dataset including diseased patients.

There has been previous work reporting fully‐automated solutions for myocardial perfusion quantification.^7^, ^8^ However, neither of these solutions at present provide a myocardial segmentation, which is the most time‐consuming manual task for the operator. The benefits of automatically segmenting the myocardium include reduced processing time in the quantification step, more interpretable parameter maps, and direct statistics for the ROI. The use of a myocardial segmentation has the potential to give a more objective diagnosis; for example, it allows the computation of the extent of perfusion defect as a percentage, which is a strong indicator of future events. Furthermore, fitting the model parameters in only the myocardium allows the use of spatial regularization^18^, ^19^ and the computation of the differences in perfusion between the endocardial and epicardial layers of the myocardium and perfusion dyssynchrony measures.^20^, ^21^

A possible alternative pipeline could have involved the individual segmentation of each timeframe in the image series. In theory, this approach would not require an explicit motion correction step, as the segmentations for each timeframe could be matched to each other. It is the success of our recently validated motion correction scheme^27^ that allows us to process just one timeframe. The benefits of this approach include that it is not necessary to design a scheme for matching points in different segmentations across different timeframes to extract voxelwise concentration curves. Moreover, it was significantly easier to gather high‐quality training data, as an observer was only asked to segment the single frame at peak LV enhancement from each slice, reducing the manual work by a factor of 100. This is likely to be important for groups that want to reproduce the pipeline. It is also a significant consideration when acquiring more data to use transfer learning to adapt the pipeline to different acquisition parameters in the future. Our approach is also likely to be more robust, as we have chosen only the timeframe with the highest SNR and contrast to process. The segmentation of all timeframes would also include precontrast frames where there is very little signal in the myocardium to guide the segmentation.

A further strength of this work is that it used a representative clinical dataset for training, including a significant proportion of diseased patients, so by default should be applicable in the clinic on data acquired using similar methods. Transfer learning techniques have already been shown to be able to account for differences in the input domain and we envisage a future application to extend the pipeline to data acquired from different types of scanners at different centers.^24^, ^25^

In our study, the size of the dataset available was limited. In order to negate this problem, data augmentation was employed. Online data augmentation was used with random transformations added to the data before each iteration of training. This helps the network to generalize better and to learn a more robust representation of the myocardium. However, this only addresses the lack of training data; it would be beneficial to further test the method on a larger dataset. A further limitation is that the primary endpoint of the analysis, the quantitative perfusion values, does not have a ground‐truth available for validation and that we have only shown that the quantitative values that are similar to those achieved manually by an expert operator. This does not investigate the diagnostic accuracy of these quantitative values, and thus further work to establish the diagnostic accuracy of the automated pipeline is warranted.

In conclusion, we proposed a fast and automated method for processing myocardial perfusion MR images prior to quantitative analysis. This automates the time‐consuming and subjective processing tasks, such as myocardial segmentation, and performs on a par with the manual experts. We anticipate that this will lead to increased adoption of quantitative perfusion analysis in the clinic as well as opening up new possibilities for research in the field.

## Conflict of Interest

M.B. is an employee of Philips Healthcare.

## Supporting information


**Appendix S1**: Supplementary material
