# Supplementary material for: Deep‐Learning‐Based Preprocessing for Quantitative Myocardial Perfusion MRI
Source: J Magn Reson Imaging. 2019 Nov 11;51(6):1689–96. doi: 10.1002/jmri.26983 (PMC7317373; doi:10.1002/jmri.26983)
Supplement: Supplementary file 1 — Appendix S1: Supplementary material [file JMRI-51-1689-s001.docx]

**Supplementary material**

**Table S1**: Demographic characteristics of the population.

|  | All  (N=175) |
| --- | --- |
| Male gender | 136 (78%) |
| Age (years) | 64.3 ± 10.3 |
| Hypertension | 86 (49%) |
| Diabetes | 34 (19%) |
| Hypercholesterolemia | 78 (45%) |
| Current / previous smoker | 24 (14%) / 18 (10%) |
| Atrial fibrillation | 42 (24%) |
| CAD status (visual assessment) | **-** |
| 1 vessel | 43 (25%) |
| 2 vessels | 28 (16%) |
| 3 vessels | 31 (18%) |

| Layer | Input size | Convolution kernel | Number of Filters |
| --- | --- | --- | --- |
| 1 | 256 x 256 | 3 x 3 | 8 |
| 2 | 128 x 128 | 3 x 3 | 16 |
| 3 | 64 x 64 | 3 x 3 | 32 |
| 4 | 32 X 32 | 3 x 3 | 64 |
| FC | 8192 | - | - |
| FC | 512 | - | - |

**Table S2.**

*The architecture used for the peak LV enhancement frame detection and bounding box detection. FC are the fully connected layers. Each convolutional layer involves convolutional with the filter with a stride length of 2 followed by batch normalisation and ReLU activation. Max-pooling is performed after every convolutional layer.*

| Layer | Input size | Convolution kernel | Number of Filters |
| --- | --- | --- | --- |
| 1-3 | 96 x 96 | 3 x 3 | 16 |
| 4-6 | 48 x 48 | 3 x 3 | 32 |
| 6-9 | 24 x 24 | 3 x 3 | 64 |
| 9-12 | 12 X 12 | 3 x 3 | 128 |
| 12-15 | 6 x 6 | 3 x 3 | 256 |
| 15-18 | 12 X 12 | 3 x 3 | 128 |
| 18-21 | 24 x 24 | 3 x 3 | 64 |
| 21-24 | 48 x 48 | 3 x 3 | 32 |
| 24-27 | 96 x 96 | 3 x 3 | 16 |

**Table S3.**

*The architecture used for the myocardial segmentation and the RV insertion point detection. On the downward trajectory (layers 1-12) each layer involves convolutional with the filter with a stride length of 2 followed by batch normalisation and ReLU activation. Max-pooling is performed after every third convolutional layer to down-sample the image dimensions. On the upward trajectory (layers 16-27), each layer still involves convolutional with the filter with a stride length of 2 followed by batch normalisation and ReLU activation with transposed convolutions being performed before every third layer to up-sample the image dimensions. Features are concatenated across the layers of the same scale, as previously described* (24,25,37)*.*

**Figure S1.**

**
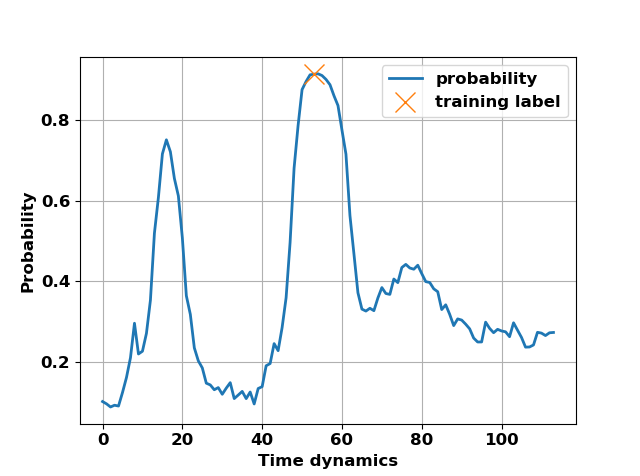
**

*The probability of being the peak LV enhancement frame over time. An increased probability is seen as contrast arrives in the LV, reaching a peak with the peak signal frame (marked with an X) and then reducing as the contrast washes out. The network also assigns relatively high probabilities to the time frames corresponding to the pre-bolus injection.*

**Figure S2.**

**
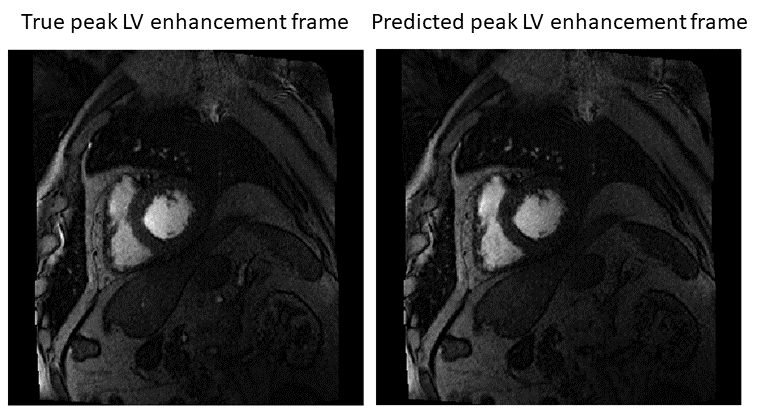
**

*A comparison of the automated and manually chosen peak LV enhancement time frame for the patient with the largest error (3 beats) in the test set. The 2 frames are virtually indistinguishable on visual assessment.*

**Figure S3.**


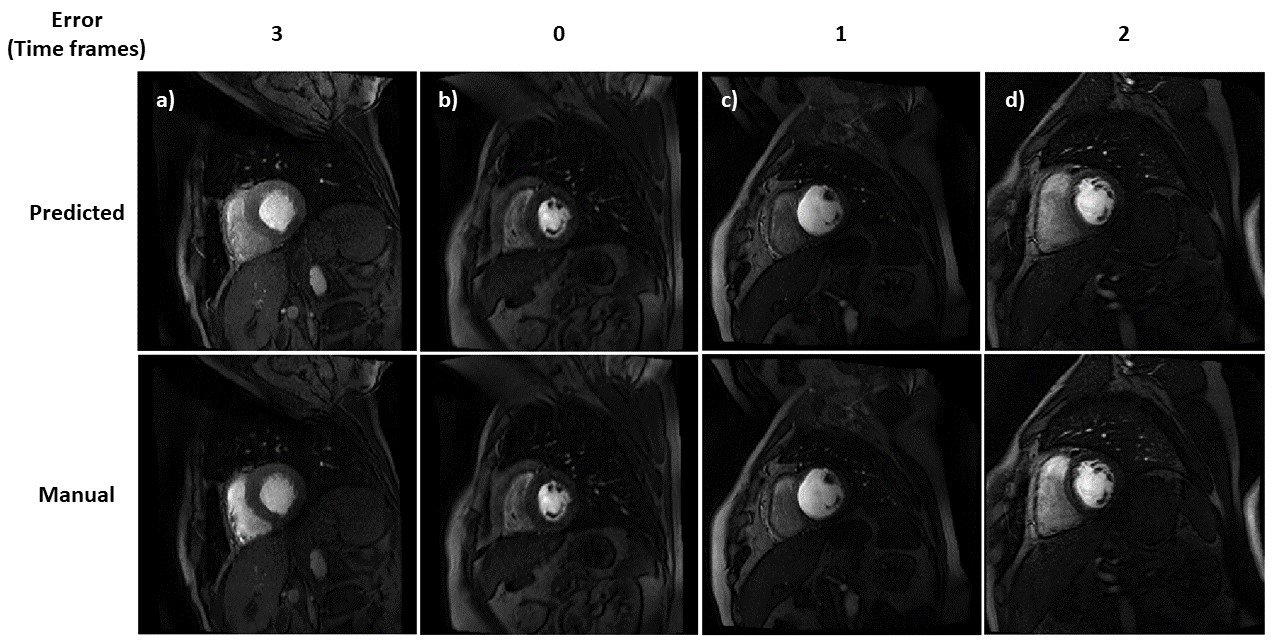


*A comparison of the automated and manually chosen peak LV enhancement time frame from a representative set of patients from the test set. The error is in terms of the number of time frames.*

**Figure S4.**


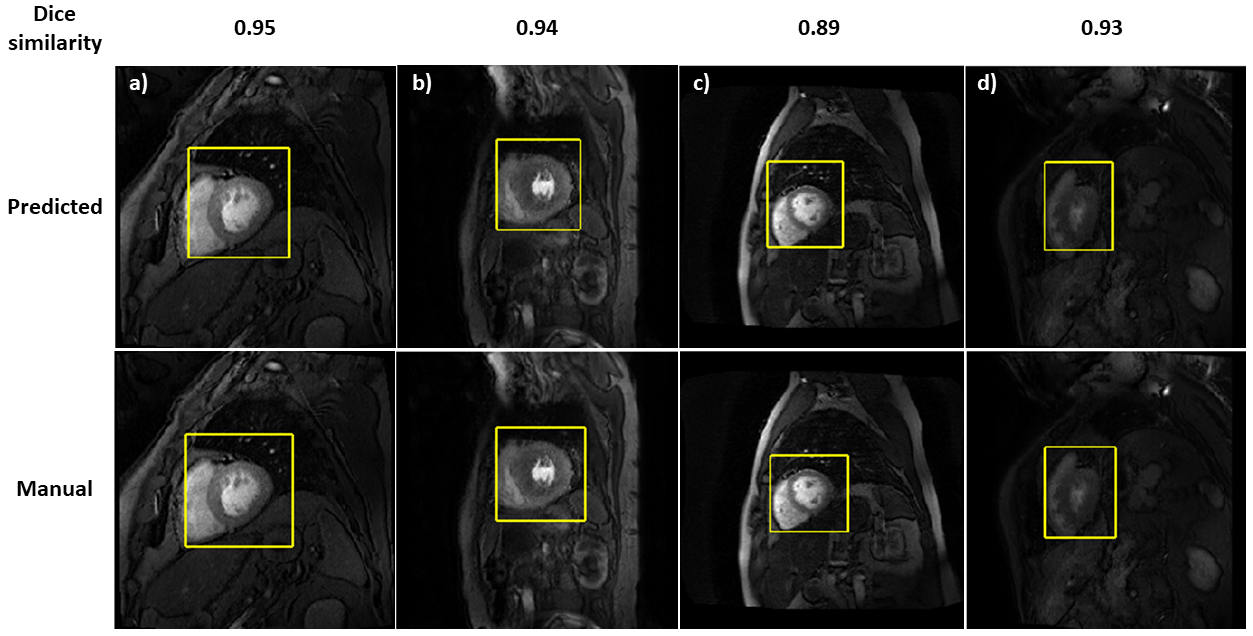


*A representative set of patients from the test set with a comparison between the automatically and manually determined bounding boxes.*

**Figure S5.**

**
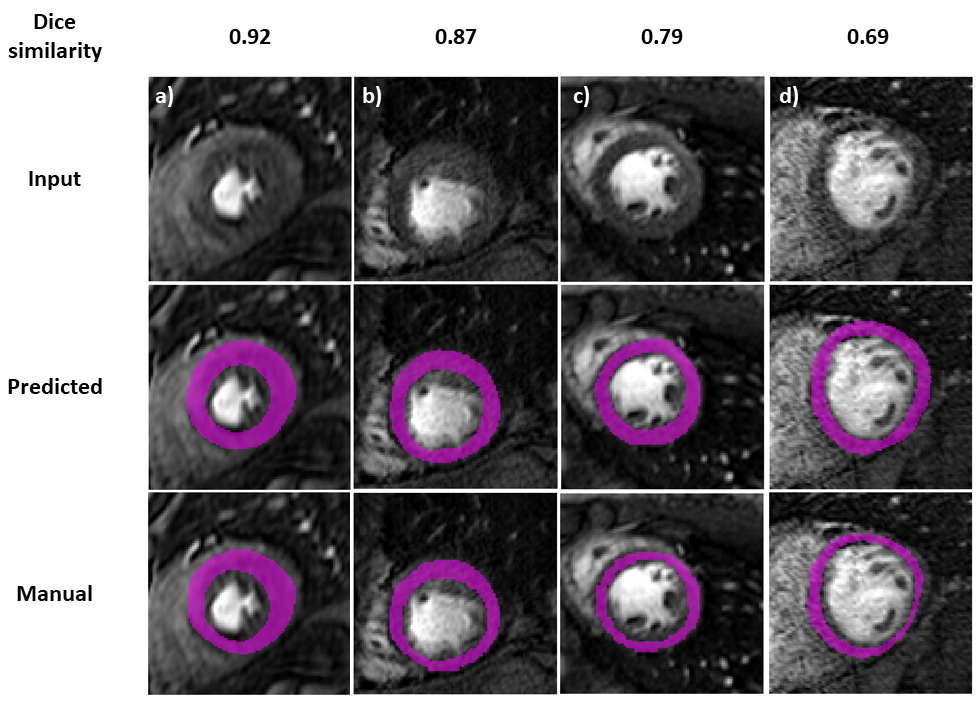
**

*A representative set of patients from the test set with a comparison between the automated segmentation and the manually defined segmentations.*

**Figure S6.**


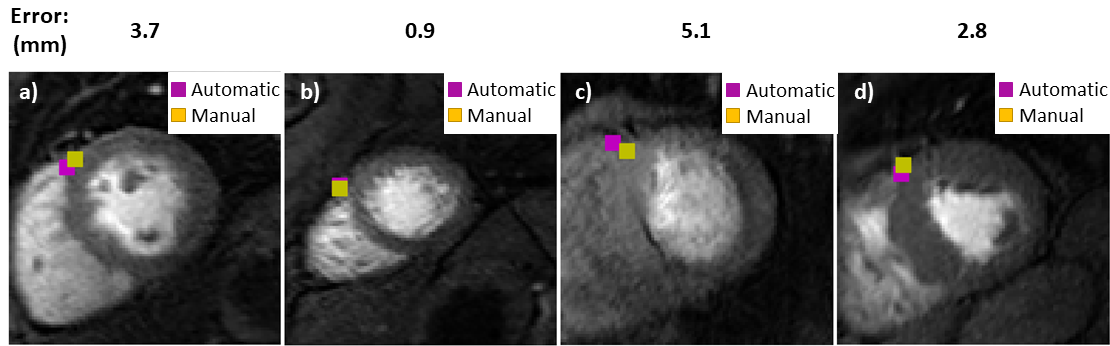


*A representative set of patients from the test set with a comparison between the automatically detected RV insertion point and the manually defined RV insertion point (error is in mm).*

**Figure S7.**

*
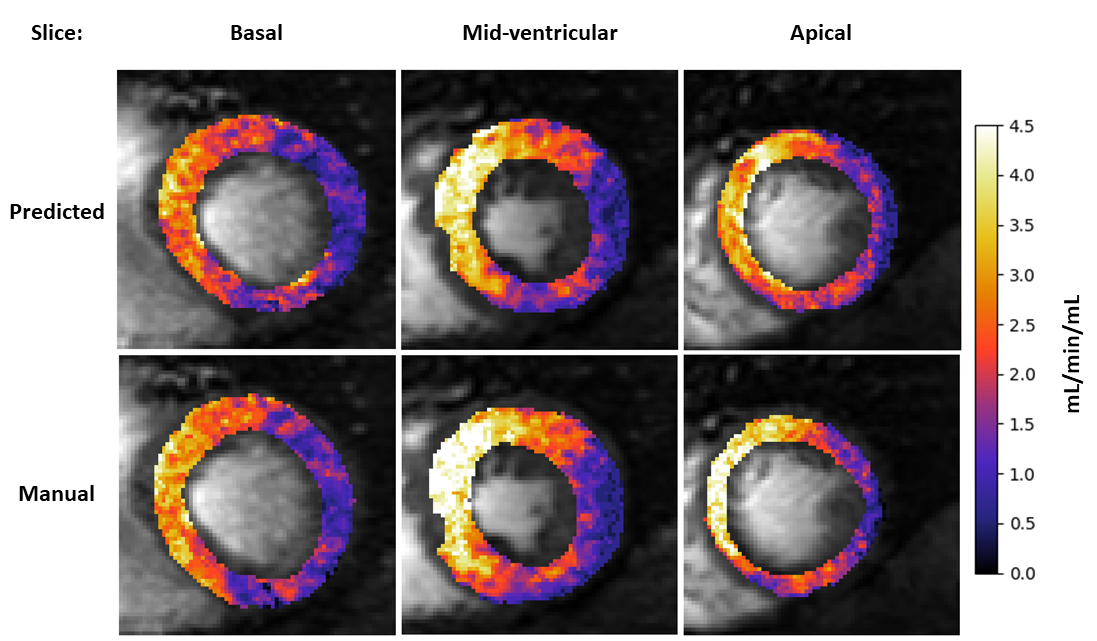
*

*A representative patient from the test set with a comparison between the automatically processed quantitative perfusion maps and the manually processed quantitative perfusion maps. Coronary angiography showed the patient has CAD with a lesion in the proximal left circumflex coronary artery*

**Figure S8.**

**
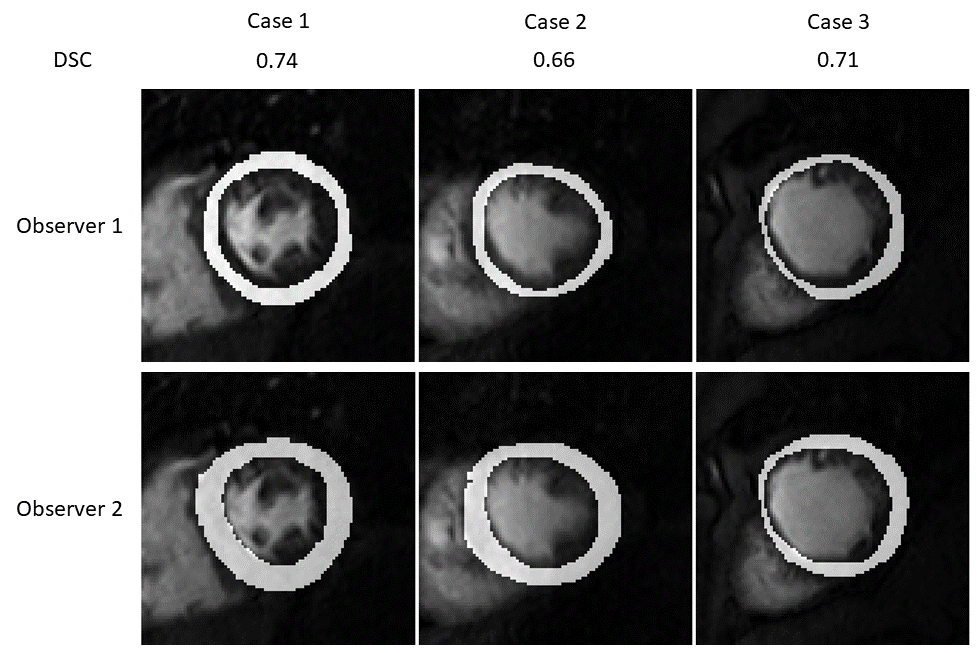
**

*A comparison between the manual segmentations obtained from two different observers.*
